# The Maternal Management of Children in Health and Disease

**Published:** 1849-08

**Authors:** 


Art. VIII.—*The Maternal Management of Children in Health and Disease.* By Thomas Bull, M. D., Member of the Royal College of Physicians, author of Hints to Mothers, etc. etc. First American from the Third London Edition. 8 vo. pp. 406. Philadelphia: Lindsay & Blackiston. 1849.

The above is the title of a work written by a London practitioner and addressed particularly to mothers.—With a large practice the author has had the best opportunities for forming a salutary code for the maternal management of children. And although we are unwilling to endorse all that he has written, still in the main he is correct and judicious. In the preface he says, "he wishes it to be distinctly remembered, that he has not written merely for those who are within reach of good medical and surgical advice, but for those also whose lot has been cast in uncivilized places, and where they are totally unable to obtain professional assistance." Hence his reason for entering into the minute details in which his book abounds.

The work is divided into two parts, the one entitled the Management of Children in Health, the other, the Management of Children in Disease. The first division contains many valuable precepts and practical suggestions touching maternal nursing, wet nursing, artificial feeding, the diet of childhood and the general management of infants and children. The second division, or the management of children in disease, is devoted to a consideration of accidents and diseases which may occur at birth and during infancy and childhood. Most of the diseases that belong to this period of life, with the remedies adapted to them are treated of, with comparatively a few unimportant exceptions, in a very pleasant, judicious and eminently practical manner. Space nor time do not, at present, allow us to make as extended a notice of the work as we wish. Before, however, taking leave of it we will transfer to our pages the experience of Dr. Bull, as found in the chapter on *Rules for the Health of the Nursing Mother* concerning the influence of mental emotions upon the lacteal secretion.

A tranquil temper, and a happy, cheerful disposition, tend greatly to promote the production of healthy milk. Indeed there is no secretion of the human body that exhibits so quickly the injurious influence of the depressing emotions as that of the breast. And although we are not able at all times to detect by any agent we possess the changes which take place in its physical properties, so delicate an apparatus for testing its qualities is the digestive system of the infant, that it will sometimes instantly show that such changes have occurred by the serious symptoms which now and then arise. I might cite many instances that I have met with illustrative of this fact. In one case, the child had had repeated fits of convulsions from noon of the previous day; the parent had been suddenly summoned to her mother, attacked with apoplexy; anxious and excited, she gave the child the breast, and within half an hour the convulsions commenced;—other breast-milk was suggested and obtained, and the fits ceased; all the previous measures had been unsuccessful. In another instance convulsions occurred evidently from disorder of the breast-milk, but the source of derangement was not discovered until a few weeks after, when it appeared that on the morning of the day when the child was attacked, the mother was made acquainted with the deranged condition of her husband's affairs. *Fear* has a powerful influence on the secretion; first changing its properties, and then frequently stopping the secretion altogether. I was sent for to an infant between two and three months old in an attack of convulsions, so severe as to threaten a fatal termination. This child I had seen at the same time the day before sucking at the breast of its wet-nurse in perfect health, never having had a moment's illness. It had shown the first symptoms of indisposition the previous night after the nurse had retired to rest; when, having been at the breast, it became restless, crying frequently, evidently from pain. In the course of the night the bowels were violently purged; towards morning the stomach would not retain the milk; and as the day advanced the general symptoms of uneasiness increased, and in the afternoon the convulsions above referred to came on. Upon inquiry I found the father of the young nurse had called on the previous evening, and not only violently abused his daughter, using severe and unwarrantable language, but had struck the poor girl, he being under the influence of liquor at the time. This interview produced such mental distress in the young woman as to attract the attention of her mistress, when an explanation of the cause ensued. Strict orders were given to forbid the man the house for the future: but the mischief was done; for it was too evident that the alarming state af the child had been produced by the deleterious change which had taken place in the nurse's milk. Remedial measures were used; the breast-milk withheld; and the infant, although it continued for many days in a doubtful state, eventually recovered: the young woman's milk, however, was altogether driven away, and another wet-nurse without delay was obliged to be obtained.

The author then cites that very remarkable instance of the effect of strong mental excitement on the secretion of the breast, mentioned by Dr. Von Ammon, which, although perhaps familiar to most of our readers, we will nevertheless quote.

"A carpenter fell into a quarrel with a soldier billeted in his house, and was set upon by the latter with his drawn sword. The wife of the carpenter at first trembled from fear and terror, and then suddenly threw herself furiously between the combatants, wrested the sword from the soldier's hand, broke it in pieces, and threw it away. During the tumult, some neighbors came in and separated the men. Whilst in this state of strong excitement, the mother took up her child from the cradle, where it lay playing, and in the most perfect health, never having had a moment's illness; she gave it the breast, and in so doing sealed its fate. In a few minutes the infant left off sucking, became restless, panted, and sank dead upon its mother's bosom. The physician who was called in found the child lying in the cradle as if asleep, and with its features undisturbed; but all his resources were fruitless. It was irrecoverably gone." The milk in this striking case must have undergone a change, which gave it a powerful sedative action upon the susceptible nervous system of the infant. *A fretful temper* will lessen the quantity of milk, make it thin and serous, and cause it to disturb the child's bowels, producing fever and griping. *Fits of anger* produce a very irritating milk, followed by griping in the infant, with green stools. *Grief or anxiety of mind* often so diminish the secretion as to render other aid necessary for the sustenance of the child. *Fear* and *terror* would seem to produce a powerful sedative effect upon the milk, as proved in Von Ammon's case just quoted, and which in a minor degree I have noticed elsewhere. A knowledge of these facts ought to serve as a salutary warning to a mother not to indulge in, but carefully to guard against, either the exciting or depessing passions.

